# Risk stratification by abbMEDS and CURB-65 in relation to treatment and clinical disposition of the septic patient at the emergency department: a cohort study

**DOI:** 10.1186/s12873-015-0056-z

**Published:** 2015-10-13

**Authors:** Asselina A. Roest, Jan Tegtmeier, Joris J. Heyligen, Jeanette Duijst, Andrea Peeters, Hella F. Borggreve, Astrid ML Oude Lashof, Coen DA Stehouwer, Patricia M. Stassen

**Affiliations:** Department of Internal Medicine, Division of General Medicine, Section Acute Medicine, Maastricht University Medical Centre, Maastricht University, Maastricht, The Netherlands; Department of Clinical Epidemiology and Medical Technology Assessment, Maastricht University Medical Centre, Maastricht University, Maastricht, The Netherlands; Department of Internal Medicine, Division of General Medicine, Section Infectious Diseases, Maastricht University Medical Centre, Maastricht University, Maastricht, The Netherlands; School CARIM, Maastricht University, Maastricht, The Netherlands; School CAPHRI, Maastricht University, Maastricht, The Netherlands

**Keywords:** Sepsis, Treatment, Clinical disposition, Risk stratification

## Abstract

**Background:**

Sepsis leads to high mortality, therefore risk stratification is important. The abbMEDS (abbreviated Mortality Emergency Department Sepsis) score assesses sepsis severity and predicts mortality. In community-acquired pneumonia, the CURB-65 (Confusion, Urea, Respiration, Blood pressure, Age) also provides support in clinical decisions regarding antibiotic treatment and clinical disposition.

We investigated the predictive value and feasibility of the abbMEDS and CURB-65 in sepsis patients at the ED and the relationship between the scores and antibiotic treatment and clinical disposition (i.e. admission and type of ward).

**Methods:**

In this retrospective cohort study, we included 725 sepsis patients at the ED. We investigated the value in predicting 28-day mortality and feasibility of both scores. We calibrated the abbMEDS. We further assessed the relationship between the three risk categories per score and antibiotic treatment (i.e. oral and intravenous narrow or broad-spectrum) and clinical disposition.

**Results:**

Both abbMEDS and CURB-65 were good predictors of 28-day mortality (13.0 %) (AUC 0.77 [95 % CI 0.72 – 0.83] and 0.73 [95 % CI 0.67 - 0.78], respectively) and feasible (complete score 92.7 and 93.9 %, respectively). In the high risk category of the abbMEDS, all patients were admitted and treated with intravenous broad-spectrum antibiotics. In the high risk category of the CURB-65, 2.5 % were not admitted and 4.4 % received no antibiotics.

**Conclusion:**

Both abbMEDS and CURB-65 are good predictors of 28-day mortality in septic ED patients. The abbMEDS is well calibrated and matches current clinical decisions concerning antibiotic treatment and clinical disposition, while this is less so for the CURB-65. In the future, use of the abbMEDS at the ED may improve sepsis care when its value as a decision support tool can be confirmed.

**Electronic supplementary material:**

The online version of this article (doi:10.1186/s12873-015-0056-z) contains supplementary material, which is available to authorized users.

## Background

Sepsis, which is defined as a systemic inflammatory response syndrome (SIRS) to an infection, is a broad clinical entity and a deadly disease [1, 2]. Sepsis can be classified as sepsis, severe sepsis or septic shock, which are associated with an increasing in-hospital mortality of16 % (sepsis) to 46 % (septic shock) [1–3]. Therefore, sepsis is an important cause of death and puts significant strain on healthcare budgets [4].

Risk stratification tools aim to assess severity of illness. In patients with community-acquired pneumonia (CAP), the CURB-65 score (Confusion, Urea, Respiration, Blood pressure, Age > 65 years) stratifies patients into three risk categories (low, intermediate, high) that predict 28-day mortality well [5]. When applied to patients with suspected infection from all foci (including CAP), the CURB-65 had good discriminatory value with regard to 28-day in-hospital mortality [6]. Besides prediction of mortality for patients with CAP, the CURB-65 provides decision support for the physician, as it advises on two management decisions, namely choice of antibiotics (e.g. small or broad-spectrum) and clinical disposition (i.e. whether a patient should be admitted to the hospital and to what type of ward) [7, 8]. The CURB-65 can therefore (in patients with CAP) be used as a decision support tool in addition to the use as an estimator of mortality risk. For patients with sepsis, the abbMEDS (Abbreviated Mortality in Emergency Department Sepsis) score, derived from the MEDS (Mortality in Emergency Department Sepsis) score has good predictive value concerning 28-day mortality [9, 10]. It would be ideal, however, if the abbMEDS could provide the same clinical guidance for sepsis as the CURB-65 does for CAP. Since there is an overlap between sepsis and CAP, we hypothesize that both the abbMEDS and CURB-65 risk categories can be related to decisions concerning management in sepsis patients.

The aim of our study was to investigate the predictive value and feasibility of the abbMEDS and CURB-65 in internal medicine patients presenting with sepsis at the ED. In addition, we aimed to assess the relationship between the three risk categories defined by both scores and two clinical decisions, i.e. empirical antibiotic treatment and clinical disposition.

## Methods

### Study design and setting

We performed a retrospective study of a cohort of patients. The Maastricht University Medical Centre (MUMC+) is a secondary and tertiary care university hospital in The Netherlands with a capacity of 715 beds. There are 30,235 ED visits per year; approximately 5000 patients are assessed by an internist. Most patients are referred by a general practitioner before visiting the ED [11]. This study was approved by the Medical Ethics Committee of MUMC+, and informed consent was deemed not to be necessary.

### Selection of participants

We reviewed the ED charts of all patients who visited the ED between 1 April 2011 and 1 August 2012 and who were assessed by an internist. Part of these patients (*n* = 704; 81.2 %) have been included in another study in which we investigated which sepsis patients were transported by ambulance [12]. We included patients above 18 years of age, who had two or more SIRS criteria and either suspected or proven infection (i.e. who fulfilled the criteria for sepsis, severe sepsis or septic shock) [1, 3]. Patients were excluded if the current admission was a re-admission within the study period (*n* = 108, 12.5 %), or when data on 28-day mortality could not be retrieved (*n* = 21, 2.4 %), despite contacting the general practitioner. Furthermore, patients were excluded when the ED chart was not complete (*n* = 7, 0.8 %) or when the patient died at the ED before the chart was completed (*n* = 1, 0.1 %).

### Methods and measurements

All data were retrieved from the ED charts and the hospital’s electronic database by the use of standardized forms and by two independent researchers. We recorded date of birth, sex, comorbidity, vital signs, laboratory results, suspected focus of infection, items of the abbMEDS and CURB-65 score (Additional file 1: Table S1), antibiotic treatment, results of microbiology cultures, clinical disposition (admission and type of ward), length of hospital stay and 28-day mortality.

Since the respiratory rate and Glasgow Coma Scale were not always exactly documented in the ED charts, we derived these items from other information in the chart in the same way as others did [6]. Items of the abbMEDS and CURB-65 were derived from patient charts. The abbMEDS and CURB-65 score were calculated and patients were assigned to the three defined risk categories (low, intermediate, high) of each score (Additional file 1: Table S1) [5, 9]. To assess the discriminatory value of both scores in the daily practice of a busy ED, only complete scores were included in our analysis with the exception of the missing abbMEDS item ‘nursing home resident’, which we imputed. This item could not always be scored (*n* = 113, 15.6 %) and we therefore made the assumption that all patients under 65 years of age (*n* = 68, 9.4 %) were not nursing home residents, unless stated otherwise. Suspected focus of infection was determined by the treating physician at the ED.

Antibiotic treatment was categorized into four groups: no antibiotics, oral antibiotics, intravenous (IV) narrow-spectrum, or IV broad-spectrum (Additional file 2: Table S2). When a patient received two narrow-spectrum antibiotics, which covered both gram positive and gram negative bacteria, we classified this as IV broad-spectrum antibiotics. Results of microbiological cultures were recorded per site (blood, urine, skin/wound and sputum). To minimize the chance of confusing infections present at the ED with nosocomial infections, we only recorded results of cultures taken at the day of presentation and two subsequent days [13, 14].

With regard to the adequacy of current empirical antibiotic treatment, we classified the choice of antibiotics started at the ED as adequate when the identified microorganisms were susceptible for the antibiotics in vitro. If a patient had a positive culture, but received no antibiotics, treatment was considered inadequate. When multiple micro-organisms were cultured, all had to be susceptible for the administered antibiotics. As coagulase-negative staphylococci in blood cultures can result from contamination, discharge letters were checked to retrieve whether the treating physician(s) regarded the culture as relevant or as contamination.

Regarding clinical disposition, all patients were stratified into three groups: no admission, admission to regular ward and medium/intensive care unit (MCU/ICU).

### Outcomes

To assess the discriminatory value of the abbMEDS and CURB-65 in predicting 28-day mortality, we plotted Receiver Operation Characteristic (ROC) curves and calculated the Area Under the Curve (AUC) with 95 % confidence intervals (CI). To calibrate the abbMEDS, we compared the observed data on 28-day mortality per risk category with those found in the derivation study of Vorwerk et al. [9]. Since the CURB-65 has only been derived in CAP patients, we did not calibrate the CURB-65 in our sepsis cohort. To assess the feasibility of the abbMEDS and CURB-65, we calculated the total number and proportion of complete scores in our study population.

We investigated two clinical decisions, i.e. the choice regarding empirical antibiotic treatment and the choice regarding clinical disposition in relation to the three abbMEDS and CURB-65 risk categories. As current empirical antibiotic treatment and clinical disposition might not be optimal, we assessed 28-day mortality for each of the groups to check for differences in mortality. To further evaluate antibiotic treatment, we investigated its adequacy in the subset of patients with positive cultures. Again, mortality was calculated for the groups with adequate and inadequate treatment.

### Analysis

We performed all statistical analyses with SPSS v.20.0 (IBM SPSS Statistics, Chicago, Illinois, USA, 2011) and MedCalc v13.3 (MedCalc Software bvba, Ostend, Belgium, 2014). Continuous data were reported as means and standard deviations or medians with interquartile ranges, categorical data as absolute counts and percentages. All percentages were valid percentages. We used the DeLong test to compare AUC values [15]. Chi-square or Fisher’s exact tests were used to test for differences in proportions. The Mann–Whitney-*U* test was used to compare medians, one way ANOVA was used to analyze trends. We analyzed mortality between the different groups of antibiotic treatment and clinical disposition with Kaplan-Meier survival analysis and the Log rank test. Associations or differences were considered statistically significant if *p* < 0.05.

## Results

### Patients

**Fig. 1:**
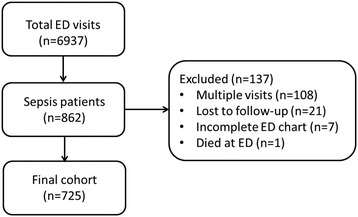
Flowchart of study population

In the 15 month study period, 6937 patients were assessed by an internist at the ED. Of these, 862 had sepsis. We excluded 137 to form a final cohort of 725 patients (Fig. 1).

In our patients, the prevalence of comorbidity was high, as 31.3 % had a malignancy and 37.5 % cardiopulmonary disease (Table 1). Ninety-four patients (13.0 %) died within 28 days after presentation. The median age was higher in non-survivors than in survivors (73.5 vs. 64.0 years, *p* < 0.0001). The abbMEDS and CURB-65 scores were higher in non-survivors than in survivors as well (*p* < 0.0001).

**Table 1:** Patient characteristics and management

n (%) or median (IQR)	All patients	Survivors	Non-survivors
	*n* = 725	*n* = 631 (87.0)	*n* = 94 (13.0)
Age, years	65.0 (53.0-77.0)	64.0 (51.0-76.0)	73.5 (63.8-83.0)*
Age >65	351 (48.4)	284 (45.0)	67 (71.3)*
Male sex	338 (46.6)	299 (47.4)	39 (41.5)
Comorbidity			
Cancer	292 (40.3)	247 (39.1)	45 (47.9)
Cardiopulmonary	233 (32.1)	189 (30.0)	44 (46.8)*
Immuno compromised	198 (27.3)	175 (27.7)	23 (24.5)
Neuropsychiatric	177 (24.4)	149 (23.6)	28 (29.8)
Diabetes mellitus	134 (18.5)	110 (17.4)	24 (25.5)
Renal disease	96 (13.2)	83 (13.2)	13 (13.8)
Liver disease	22 (3.0)	21 (3.3)	1 (1.1)
Suspected focus of infection			
Lower respiratory tract	213 (29.4)	179 (28.4)	34 (36.2)
Urinary tract	152 (21.0)	138 (21.9)	14 (14.9)
Gastrointestinal tract	94 (13.0)	82 (13.0)	12 (12.8)
Hepatobiliary system	43 (5.9)	37 (5.9)	6 (6.4)
Skin	39 (5.4)	34 (5.4)	5 (5.3)
Upper respiratory tract	37 (5.1)	36 (5.7)	1 (1.1)
Other	147 (20.3)	125 (19.8)	22 (23.4)
Antibiotics at ED	647 (89.2)	558 (88.4)	89 (94.7)
Disposition			
No admission	63 (8.7)	62 (9.8)	1 (1.1)†
Regular ward	626 (86.3)	544 (86.2)	82 (87.2)
MCU/ICU	36 (5.0)	25 (4.0)	11 (11.7)‡
Length of hospital stay, days	6 (3–11)	7 (3–12)	5 (2–10)‡
Severity scores			
AbbMEDS	5 (2–8)	3 (2–7)	8 (6–11)*
CURB-65	1 (0–2)	1 (0–2)	2 (1–3)*

*SD* Standard Deviation, *MCU* Medium Care Unit, *ICU* Intensive Care Unit, *IQR* Interquartile Range

*P < 0.0001, †*P* < 0.01, ‡*P* < 0.05

### Discriminatory value

**Fig. 2:**
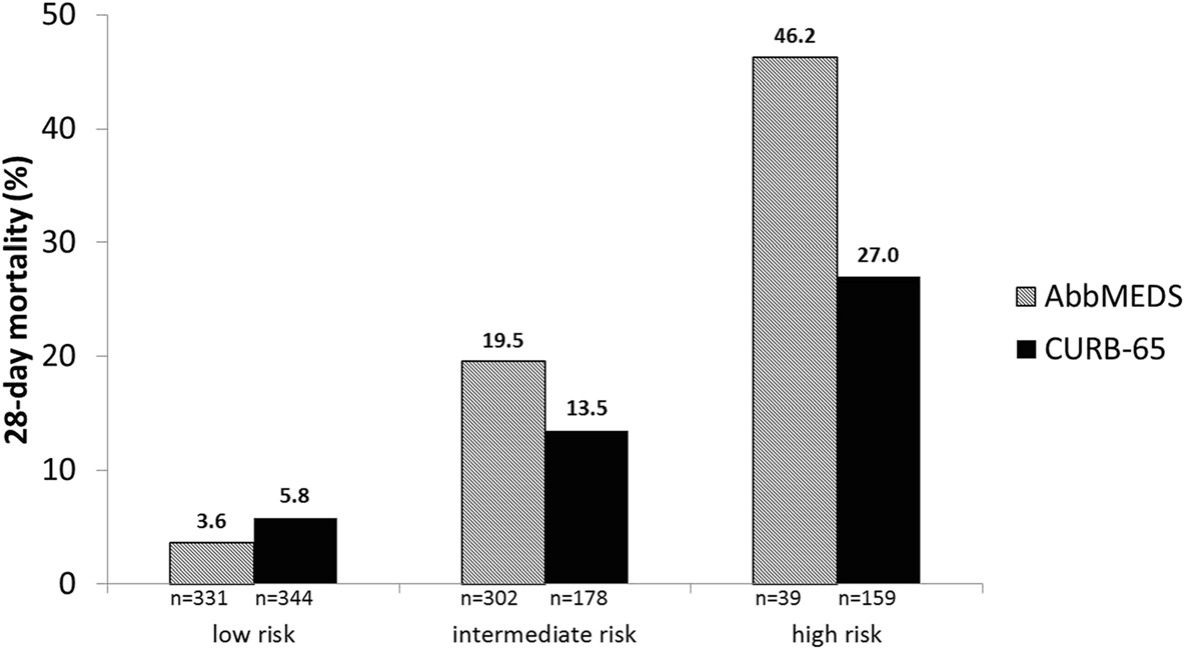
Number of patients and 28-day mortality for the three risk categories of abbMEDS and CURB-65

Mortality increased per increasing risk category for both scores (Fig. 2). Compared to the abbMEDS, the CURB-65 assigned more patients to the high risk category (5.8 vs. 23.3 %). The AUC of the abbMEDS was 0.77 [95 % CI: 0.72–0.83] and that of the CURB-65 0.73 [95 % CI: 0.67–0.78] for predicting 28-day mortality. This difference was not statistically significant (*p* = 0.08).

### Feasibility

Complete risk scores could be calculated in 672 (92.7 %) patients for the abbMEDS, and in 681 (93.9 %) for the CURB-65 (*p* = 0.40). The abbMEDS was incomplete due to missing information concerning the item “nursing home resident” (*n* = 45, 6.2 % (>65 years)) or missing thrombocytes (*n* = 8, 1.1 %). The CURB-65 score was incomplete mostly due to missing urea values (*n* = 42, 5.8 %). The survival of those with complete and incomplete scores was not different (*p* = 0.42).

### Calibration

Table 2 shows that mortality increased per increasing abbMEDS risk category in parallel with the mortality found in the derivation study by Vorwerk et al. [9]. In our study, mortality in the low risk category was higher than in the derivation study (3.6 vs. 1.6 %), while in the intermediate and high risk categories our mortality was lower (19.5 vs. 23.4 % and 46.2 vs. 59.0 %, respectively).

**Table 2:** Mortality within 28 days per abbMEDS risk category compared to the derivation study (10)

Risk category	28-day mortality, n (%)			
	Current study		Derivation study (10)	
	Mortality	Patients	Mortality	Patients
Low risk	12 (3.6)	331	1 (1.6)	63
Intermediate risk	59 (19.5)	302	48 (23.4)	205
High risk	18 (46.2)	39	23 (59.0)	39
Total	89 (13.2)	672	72 (23.5)	307

### Antibiotic treatment in relation to risk categories

Of the 725 patients, 78 (10.8 %) did not receive antibiotic treatment, 71 (9.8 %) received oral and 34 (4.7 %) IV narrow-spectrum antibiotics and the majority were treated with IV broad-spectrum antibiotics (74.7 %, Table 3). In the low and intermediate risk categories of both scores, antibiotic treatment was almost similar. In contrast, all high risk abbMEDS patients were treated with IV broad-spectrum antibiotics, while 15 (9.4 %) high risk CURB-65 patients were treated with no, oral or IV narrow-spectrum antibiotics. There were no significant differences in mortality between the four groups of antibiotic treatment in the intermediate risk category of both scores, but the numbers were too small to draw firm conclusions. Mortality in the four groups of antibiotic treatment in the low and high risk categories were not compared with each other, because in some subgroups nobody died.

**Table 3:** Antibiotic treatment and 28-day mortality per risk category of the abbMEDS and CURB-65

n (%)	Low risk		Intermediate risk		High risk	
	Patients	Mortality	Patients	Mortality	Patients	Mortality
AbbMEDS						
No antibiotics	52 (15.7)	1 (1.9)	20 (6.6)	4 (20.0)	0	0
Oral	41 (12.4)	0	21 (7.0)	3 (14.3)	0	0
IV narrow-spectrum	22 (6.6)	0	10 (3.3)	1 (10.0)	0	0
IV broad-spectrum	216 (65.3)	11 (5.1)	251 (83.1)	51 (20.3)	39 (100.0)	18 (46.2)
Total (672)#	331 (49.3)	12 (3.6)	302 (44.9)	59 (19.5)	39 (5.8)	18 (46.2)
CURB-65						
No antibiotics	54 (15.7)	3 (5.6)	10 (5.6)	0	7 (4.4)	1 (14.3)
Oral	47 (13.7)	3 (6.4)	13 (7.3)	1 (7.7)	4 (2.5)	0
IV narrow-spectrum	24 (7.0)	0	5 (2.8)	0	4 (2.5)	0
IV broad-spectrum	219 (63.7)	14 (6.4)	150 (84.3)	23 (15.3)	144 (90.6)	41 (28.5)
Total (681)#	344 (50.5)	20 (5.8)	178 (26.1)	24 (13.5)	159 (23.3)	42 (26.4)

#The totals are depicted to show how many data were available for all patients

There were no significant differences in mortality per treatment class within each risk category

### Adequacy of empirical antibiotic treatment in relation to risk categories

One or more positive culture(s) were found in 244 patients (Additional file 3: Table S3). Seventeen patients were excluded because their cultures were considered to result from contamination. Empirical antibiotic treatment was considered adequate in 169 (74.4 %) of the remaining 227 patients (Table 4). There was no significant difference in 28-day mortality between the groups with adequate (13.6 %) and inadequate treatment (12.1 %, *p* = 0.48, Log rank test). The higher the abbMEDS risk category, the more frequent antibiotic treatment was considered adequate (*p* = 0.04). For the CURB-65, adequacy of antibiotic treatment was not different between the three risk categories (*p* = 0.09).

**Table 4:** Adequacy of antibiotic treatment and 28-day mortality per risk category of the abbMEDS and CURB-65

n (%)	Low risk		Intermediate risk		High risk	
	Patients	Mortality	Patients	Mortality	Patients	Mortality
AbbMEDS						
Adequate*	60 (65.9)	1 (1.7)	80 (80.8)	13 (16.3)	13 (92.9)	6 (46.2)
Inadequate	31 (34.1)	1 (3.2)	19 (19.2)	5 (26.3)	1 (7.1)	1 (100)
Total (204)#	91 (44.6)	2 (2.2)	99 (48.5)	18 (18.2)	14 (6.9)	7 (50.0)
CURB-65						
Adequate	61 (70.1)	4 (6.6)	43 (70.5)	3 (7.0)	58 (84.1)	15 (25.9)
Inadequate	26 (29.9)	1 (3.8)	18 (29.5)	5 (27.8)	11 (15.9)	1 (9.1)
Total (217)#	87 (40.1)	5 (5.7)	61 (29.5)	8 (13.1)	69 (31.8)	16 (23.2)

#The totals are depicted to show how many data were available for all patients

*p = 0.04 for trend. Adequacy was defined as in vitro susceptibility of the identified microorganisms for the empirical treatment

### Clinical disposition in relation to risk categories

Across the three risk categories of both scores, the majority of patients were admitted to the hospital (Table 5). The main difference between both scores was that all high risk abbMEDS patients were admitted, while four (2.5 %) high risk CURB-65 patients were not admitted. The proportion of patients, who were treated as outpatients, was highest in the low risk category and almost equal for both the abbMEDS and CURB-65. The only patient in the intermediate risk category of the abbMEDS who was treated as an outpatient, had terminal cancer and went home on his own request, where he died. None of the CURB-65 patients, treated as outpatients, died. While most patients were admitted to a regular ward, the percentage of patients admitted to a MCU/ICU increased through the risk categories in both scores to 12.6 and 11.9 % in the high risk category of abbMEDS and CURB-65, respectively.

**Table 5:** Clinical disposition and 28-day mortality per risk category of the abbMEDS and CURB-65

n (%)	Low risk		Intermediate risk		High risk	
	Patients	Mortality	Patients	Mortality	Patients	Mortality
AbbMEDS						
No admission	40 (12.1)	0	16 (5.3)	1 (6.3)	0	0
Regular ward	289 (87.3)	12 (4.2)	259 (85.8)	50 (19.3)	34 (87.2)	15 (44.1)
MCU/ICU	2 (0.6)	0	27 (8.9)	8 (29.6)	5 (12.8)	3 (60.0)
Total (672)#	331 (49.3)	12 (3.6)	302 (44.9)	59 (19.5)	39 (5.8)	18 (46.2)
CURB-65						
No admission	46 (13.4)	0	5 (2.8)	0	4 (2.5)	0
Regular ward	295 (85.8)	19 (6.4)	161 (90.4)	20 (12.4)	136 (85.5)	38 (27.9)
MCU/ICU	3 (0.9)	1 (33.3)	12 (6.7)	4 (33.3)	19 (11.9)	5 (26.3)
Total (681)#	344 (50.5)	20 (5.8)	178 (26.1)	24 (13.5)	159 (23.3)	43 (27.0)

# The totals are depicted to show how many data were available for all patients

There were no significant differences in mortality per disposition group within each risk category

## Discussion

This study investigated the value of abbMEDS and CURB-65 in internal medicine patients presenting with sepsis at the ED. We found a moderate predictive value regarding 28-day mortality of both the abbMEDS (AUC 0.77) and CURB-65 (AUC 0.73). In addition, both scores were feasible at our ED with complete scores in 92.7 and 93.9 %, respectively. New findings are that the abbMEDS categorizes patients well with respect to two important clinical decisions, namely empirical antibiotic treatment (e.g. narrow or broad-spectrum) and clinical disposition (i.e. whether a patient is admitted to the hospital and to what level of care), and that the abbMEDS performs better than the CURB-65.

With regard to the discriminatory value of the AbbMEDS, we found a slightly lower AUC than in the derivation study (AUC 0.77 vs. 0.82) by Vorwerk et al. [9], which may result from a difference in patient selection. The abbMEDS was well calibrated in our population, but compared to the derivation study, we found a higher mortality in the low risk category (3.6 vs. 1.6 %). An explanation for this finding may be that, due to the prominent role of the general practitioner in the Netherlands, the patients who are referred to the hospital and then assigned to the low risk category are sicker than patients in other countries [11, 16]. The CURB-65 was developed for patients with CAP [5]. Later, the CURB-65 was validated in an ED population with an infection [6]. In that study, a higher AUC (0.79) was found than in ours (0.73), which might be explained by the higher mortality in our study population (13.0 vs. 3.9 %). The mortality in our study was higher because we selected patients with sepsis instead of patients with infection.

The first new finding of our study is that the abbMEDS matches two current clinical decisions in the management of sepsis patients, namely empirical antibiotic treatment and clinical disposition. This is less the case for the CURB-65. In the low risk categories of both scores, roughly 30 % did not receive antibiotics or were treated with oral antibiotics (i.e. treatment that would not require admission to the hospital). The most severely ill patients in the high risk categories received almost exclusively IV broad-spectrum antibiotics. This is where the abbMEDS matches current clinical decisions better than the CURB-65, for all high risk abbMEDS patients were treated with IV broad-spectrum antibiotics, in contrast to 9.4 % of the high risk CURB-65 patients who were treated with no or oral antibiotics. We did not find significant differences in mortality between these different treatment groups. However, our mortality figures are too small to draw conclusions on this matter. We additionally analyzed adequacy of our empirical treatment (antibiotics started at the ED) based on in vitro susceptibility. We found that adequacy was related to the abbMEDS risk categories. With increasing risk category of the abbMEDS, more patients were treated with adequate antibiotics. However, this was not the case for CURB-65, as 15.9 % of the patients in the high risk category received inadequate treatment. The need for high adequacy is most urgent in the high risk category with the most severely ill patients [17]. For the less severely ill patients, a lower adequacy can be accepted, as confining microbial resistance to antibiotics is more important in this large group of patients.

The second new finding regarding clinical decisions was that the abbMEDS matches current management regarding clinical disposition. Again, the CURB-65 did this less well. With increasing risk category of the abbMEDS, more patients were admitted to the MCU/ICU, while the fraction of patients who were not admitted to the hospital decreased. In contrast, in the high risk category of the CURB-65, 4 (2.5 %) patients were not admitted.

To our knowledge, we showed for the first time that the abbMEDS matches current clinical decisions regarding empirical antibiotic treatment and clinical disposition of sepsis patients at the ED. In patients with CAP, the CURB-65 is already used to provide clinical decision support. This resulted in more standardized care and reduced the use of broad-spectrum antibiotics, while patient safety was maintained [18, 19]. We think that the abbMEDS has the potential to provide clinical decision support in patients presenting with sepsis. However, future studies are needed and these should confirm that the abbMEDS can indeed appropriately and safely be used to provide this support on clinical decisions. The abbMEDS could then develop from a risk stratification score into a clinical decision support tool with the potential to increase the standard of care in sepsis patients. Hopefully, this will yield improved survival, reduced use of broad-spectrum antibiotics and reduced healthcare costs in sepsis patients.

This study has some limitations. First, we cannot exclude the risk of misclassification of measurements or data. However, we used standardized extraction forms and the main factors studied (laboratory values, antibiotic treatment, clinical disposition and mortality) are less likely to be subject of misclassification. Second, our study was limited to one hospital, but we included over 700 patients in a 15-month study period. Third, current clinical decisions might not be optimal as they are physician-dependent. However, our study reflects clinical practice and it is improbable that our management is less optimal than in other hospitals. A way to examine adequate management in a better way is to ask a panel of experts whether they agree with the management decisions based on the calculated score in selected cases. Considering the limitations, our study must be seen as an exploratory one. To our opinion, the results justify a prospective study that implements such a panel.

## Conclusions

Both the abbMEDS and CURB-65 are good predictors of 28-day mortality in sepsis patients and are very feasible at the ED. The abbMEDS is well calibrated. A new finding is that the abbMEDS matches current clinical decisions in sepsis patients concerning antibiotic treatment and clinical disposition well, while this is less the case for the CURB-65. To improve the standard of care of sepsis, further research on the ability of the abbMEDS to provide support in clinical decisions in sepsis patients at the ED must be performed.

## Additional files

Additional file 1: Table S1. The abbMEDS (left panel) and CURB-65 score (right panel) with risk categories. Description of data: Items and risk categories of abbMEDS and CURB-65. (PDF 68 kb) Additional file 2: Table S2. Classification of antibiotics. Description of data: Classification of antibiotic treatment in three groups; oral, IV narrow-spectrum and IV-broad spectrum. (PDF 9 kb) Additional file 3: Table S3. Positive cultures per site and most commonly identified pathogens. Description of data: Description of positive cultures per site and the most commonly identified pathogens. (PDF 7 kb)
