# Supplementary material for: Risk stratification by abbMEDS and CURB-65 in relation to treatment and clinical disposition of the septic patient at the emergency department: a cohort study
Source: BMC Emerg Med. 2015 Oct 13;15:29. doi: 10.1186/s12873-015-0056-z (PMC4605126; doi:10.1186/s12873-015-0056-z)
Supplement: Additional file 1: Table S1. — The abbMEDS (left panel) and CURB-65 score (right panel) with risk categories. Description of data: Items and risk categories of abbMEDS and CURB-65. (PDF 68 kb) [file 12873_2015_56_MOESM1_ESM.pdf]

Additional Table 1: The abbMEDS (left panel) and CURB-65 score (right panel) with risk categories [7,10]

| **abbMEDS item** | **Score** | **CURB-65 item** | **Score** |
|---|---|---|---|
| Terminal disease | 6 | Confusion | 1 |
| Respiratory difficulty | 3 | Urea > 7 mmol/l | 1 |
| Septic shock | 3 | Respiratory rate > 30/min | 1 |
| Thrombocytes < $150x10^9$ cells/liter | 3 | Blood pressure (SBP < 90 or DBP ≤ 60 mmHg) | 1 |
| Age > 65 years | 3 | Age ≥ 65 years | 1 |
| Lower respiratory tract infection | 2 | | |
| Nursing home resident | 2 | | |
| Altered mental state | 2 | | |
| **Total** | **24** | **Total** | **5** |
| **Risk category** | **Score** | **Risk category** | **Score** |
| low risk | 0 – 4 | low risk | 0 – 1 |
| intermediate risk | 5 – 12 | intermediate risk | 2 |
| high risk | 13 – 24 | high risk | 3 – 5 |
