# Supplementary material for: Risk stratification by abbMEDS and CURB-65 in relation to treatment and clinical disposition of the septic patient at the emergency department: a cohort study
Source: BMC Emerg Med. 2015 Oct 13;15:29. doi: 10.1186/s12873-015-0056-z (PMC4605126; doi:10.1186/s12873-015-0056-z)
Supplement: Additional file 2: Table S2. — Classification of antibiotics. Description of data: Classification of antibiotic treatment in three groups; oral, IV narrow-spectrum and IV-broad spectrum. (PDF 9 kb) [file 12873_2015_56_MOESM2_ESM.pdf]

Additional Table 2: Classification of antibiotics

Oral	IV narrow-spectrum	IV broad-spectrum
Amoxicillin	Amoxicillin	Amoxicillin/clavulanate
Amoxicillin/clavulanate	Ciprofloxacin	Amoxicillin/clavulanate + gentamicin
Ciprofloxacin	Cotrimoxazole	Cefalozin
Cotrimoxazole	Flucloxacillin	Cefotaxime
Doxycycline	Penicillin	Ceftazidime
Flucloxacillin	Clindamycin	Ceftriaxone
Nitrofurantoin		Cefuroxime
		Ciprofloxacin + flucloxacillin
In case of allergy / contra indication:		Ciprofloxacin + gentamicin
Azithromycin		Clindamycin + ciprofloxacin
Clarithromycin		Levofloxacin
Erythromycin		Meropenem
		Moxifloxacin
		Piperacillin/tazobactam
		Vancomycin + ciprofloxacin
