# Supplementary material for: Risk stratification by abbMEDS and CURB-65 in relation to treatment and clinical disposition of the septic patient at the emergency department: a cohort study
Source: BMC Emerg Med. 2015 Oct 13;15:29. doi: 10.1186/s12873-015-0056-z (PMC4605126; doi:10.1186/s12873-015-0056-z)
Supplement: Additional file 3: Table S3. — Positive cultures per site and most commonly identified pathogens. Description of data: Description of positive cultures per site and the most commonly identified pathogens. (PDF 7 kb) [file 12873_2015_56_MOESM3_ESM.pdf]

Supplemental Table 3: Positive cultures per site and most commonly identified pathogens

Site of culture	n (%)		Most common pathogen (%)	
Urine	155	(48.9)	Escherichia coli	(61.8)
Blood	98	(30.9)	Escherichia coli	(32.7)
Wound/skin	42	(13.2)	Staphylococcus aureus	(28.6)
Sputum	22	(6.9)	Haemophilus influenzae	(27.3)
